# Predicting circRNA-Disease Associations Based on circRNA Expression Similarity and Functional Similarity

**DOI:** 10.3389/fgene.2019.00832

**Published:** 2019-09-12

**Authors:** Yongtian Wang, Chenxi Nie, Tianyi Zang, Yadong Wang

**Affiliations:** School of Computer Science and Technology, Harbin Institute of Technology, Harbin, China

**Keywords:** circRNA, disease, circRNA expression similarity, circRNA functional similarity, PersonalRank

## Abstract

Circular RNAs (circRNAs) are a novel class of endogenous noncoding RNAs that have well-conserved sequences. Emerging evidence has shown that circRNAs can be novel biomarkers or therapeutic targets for many diseases and play an important role in the development of various pathological conditions. Therefore, identifying potential disease-related circRNAs is helpful in improving the efficiency of finding therapeutic targets for diseases. Here, we propose a computational model (PreCDA) to predict potential circRNA–disease associations. First, we calculated the circRNA expression similarity based on circRNA expression profiles. The circRNA functional similarity is calculated based on cosine similarity, and the disease similarity is used as the dimension of each circRNA vector. The associations between circRNAs and diseases are defined based on the circRNA functional similarity and expression similarity. We constructed a disease-related circRNA association network and used a graph-based recommendation algorithm (PersonalRank) to sort candidate disease-related circRNAs. As a result, PreCDA has an average area under the receiver operating characteristic curve value of 78.15% in predicting candidate disease-related circRNAs. In addition, we discuss the factors that affect the performance of this method and find some unknown circRNAs related to diseases, with several common diseases used as case studies. These results show that PreCDA has good performance in predicting potential circRNA–disease associations and is helpful for the diagnosis and treatment of human diseases.

## Introduction

Circular RNAs (circRNAs) are a type of RNA molecule that forms a covalently closed continuous loop from exon circularization (Motieghader et al., 2017; Xu, 2017). In recent years, advances in high-throughput sequencing technology have greatly facilitated the study of circRNAs (Jeck and Sharpless, 2014). When compared to other ncRNAs (Danan et al., 2012), circRNAs are highly stable. Circular RNAs have evolutionarily conserved sequence features across species, tissues, and developmental stages (Jens, 2013; Conn et al., 2015; Rybak-Wolf et al., 2015). Therefore, circRNAs have become hotspots in transcriptomics research.

Recent studies have shown that alterations in the expression of circRNAs play important roles in human disease and other biological processes (Xu, 2017; Zhao and Shen, 2017; Xia et al., 2018). For example, the best-known circRNA, CDR1as, as the inhibitor of miR-7, is a critical ncRNA known to be involved in cancer, neurodegenerative diseases, diabetes, and atherosclerosis (Li et al., 2015; Xu et al., 2018). Researchers found that the circRNA ciRS-7 may be a promising target for neurodegenerative disorder (Lukiw, 2013) and myocardial infarction (Lin et al., 2018). The circRNA CircCCDC66 has been demonstrated to regulate colon cancer growth and metastasis as a miRNA sponge (Hsiao et al., 2017). The circRNA hsa_circ_0001895 is involved in the expression of cancer-related proteins in gastric cancer (Shao et al., 2017). The circRNA CircHIPK3 plays an important role in cell growth by sponging multiple miRNAs (Zheng et al., 2016). Moreover, circRNAs can be found in exosomes, cell-free saliva, and plasma (Li Y et al., 2015). Circular RNAs are emerging as novel biomarkers or therapeutic targets for many diseases due to their conservation, cell type–specific expression, and tissue-specific expression, and they play roles in the development of various pathological conditions (Meng et al., 2017; Vo et al., 2018).

Although a large number of circRNAs have been discovered, the mechanisms of circRNAs in many diseases remain unclear (Xu et al., 2018). To enable research on circRNAs and diseases, several databases have been constructed, such as circRNADisease (Zhao et al., 2018), CircR2Disease (Fan et al., 2018), and Circ2Disease (Yao et al., 2018). They provide important data support for circRNA–disease association analyses. Some methods have been proposed to provide the most promising disease-related biomarkers, including those involving lncRNAs (Chen et al., 2015; Gu et al., 2017; Cheng et al., 2018a; Cheng et al., 2019), miRNAs (Peng et al., 2019b; Shao et al., 2018), genes (Cheng et al., 2016; Hu et al., 2019; Peng et al., 2019a), and drugs (Jiang et al., 2017; Zhang et al., 2017), for further experimental validation. These methods can decrease the time and cost of biological experiments. However, very few methods have been developed to predict potential circRNA–disease associations (Lei et al., 2018), and both disease functional similarity and semantic similarity were not considered in these methods. Improved knowledge has suggested that exploring both the semantic and functional associations of diseases, which are two types of significant associations, is beneficial in measuring disease similarity (Cheng et al., 2014; Peng et al., 2018).

In this study, we proposed a computational model (PreCDA) for potential disease-related circRNA identification. In view of the limited number of circRNA–disease associations, we introduced disease similarity to solve possible sparse problems and built a disease-related circRNA similarity network. However, relying entirely on circRNA-related diseases greatly limits the utility of the method because many circRNAs still have very few or no associated diseases. To overcome this limitation, we calculated the circRNA expression similarity based on the existing data resources. Subsequently, we built a new disease-associated circRNA network by fusing circRNA functional associations and expression similarities. To assess the practicability and accuracy of this method, we designed a validation process with different datasets of circRNA–disease associations, as good computational models must perform well on different data sources. Finally, PreCDA proved successful in predicting potential disease-related circRNAs.

## Materials and Methods

### Workflow

**Figure 1:**
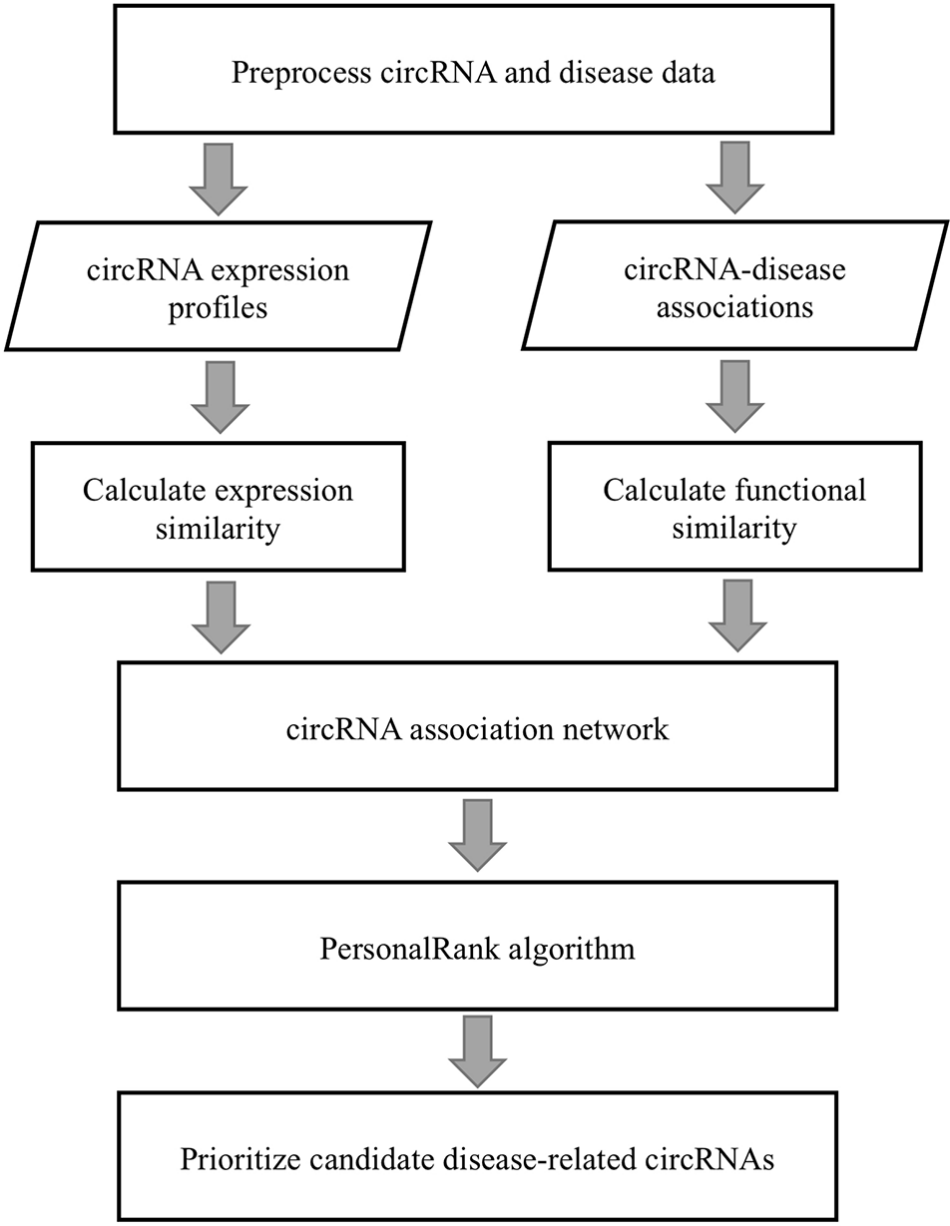
Flowchart of the PreCDA workflow.

A flowchart of the PreCDA workflow is shown in **Figure 1**. We preprocessed circRNA and disease data because of the lack of uniform identification of circRNAs and diseases. We extracted the synonym vocabulary from the two circRNA databases, including circRNADisease (Zhao et al., 2018) and circBase (Glažar et al., 2014). Then, we unified different representations of the same circRNA in different databases. Additionally, the identification of the Human Disease Ontology (DO) (Kibbe et al., 2015) was used as the unified marker of diseases in the computational model. We measured the similarity between circRNAs in two ways, including the circRNA expression similarity and functional similarity. We extracted circRNA expression profiles from circBase (Glažar et al., 2014) and CIRCpedia (Dong et al., 2018). The circRNA expression similarity was calculated based on the Spearman correlation coefficient. The disease similarity was used as the dimension of each circRNA vector, and the circRNA functional similarity was calculated based on cosine similarity. A disease-related circRNA association network was built based on the circRNA expression similarity and functional similarity. Finally, we identified potential candidate disease-related circRNAs based on the PersonalRank algorithm (PR) (Haveliwala, 2002).

### Data Preprocessing

#### circRNA Data

In this study, we used three circRNA databases for experiments and validations. The circRNADisease database is a manually curated database of experimentally supported circRNA and disease associations, which collected 330 circRNAs and 48 diseases in 354 associations. Each entry in the circRNADisease database includes detailed information on a circRNA–disease association, including the circRNA and disease name, the circRNA expression pattern, literature references, and other annotation information. CircR2Disease is a database for experimentally supported circRNA–disease associations and provides a platform for investigating the mechanism of disease-related circRNAs. The present version of CircR2Disease collected 661 circRNAs and 100 diseases. Circ2Disease is a database that curates experimentally supported human circRNAs and provides comprehensive associations between circRNAs and human diseases. It contains 273 manually curated associations between 237 circRNAs and 54 human diseases from 120 studies. However, currently, the naming of circRNAs has not yet been unified (Xu et al., 2018), which leads to the underutilization of information from different public circRNA databases. Therefore, we designed and collected mappings among different circRNA names provided by different circRNA databases, including circRNADisease and circBase. circRNADisease contains circRNA synonyms, and circBase is a database that merged and unified datasets of circRNAs. We mapped circRNAs from the three circRNA databases to circBase referring to circRNA synonyms. Then, we used circRNA IDs from circBase as the unified IDs of circRNAs in this work.

#### Disease Data

Human Disease Ontology represents common and rare human disease concepts captured across biomedical resources. Each node in DO represents one disease term and is organized in a directed acyclic graph with the relationship of “is_a”. MEDIC (Davis et al., 2012) integrates OMIM (Online Mendelian Inheritance in Man) terms (Amberger et al., 2015), synonyms and identifiers with MeSH terms (Lipscomb, 2000), synonyms, definitions, identifiers, and hierarchical relationships.

**Figure 2:**
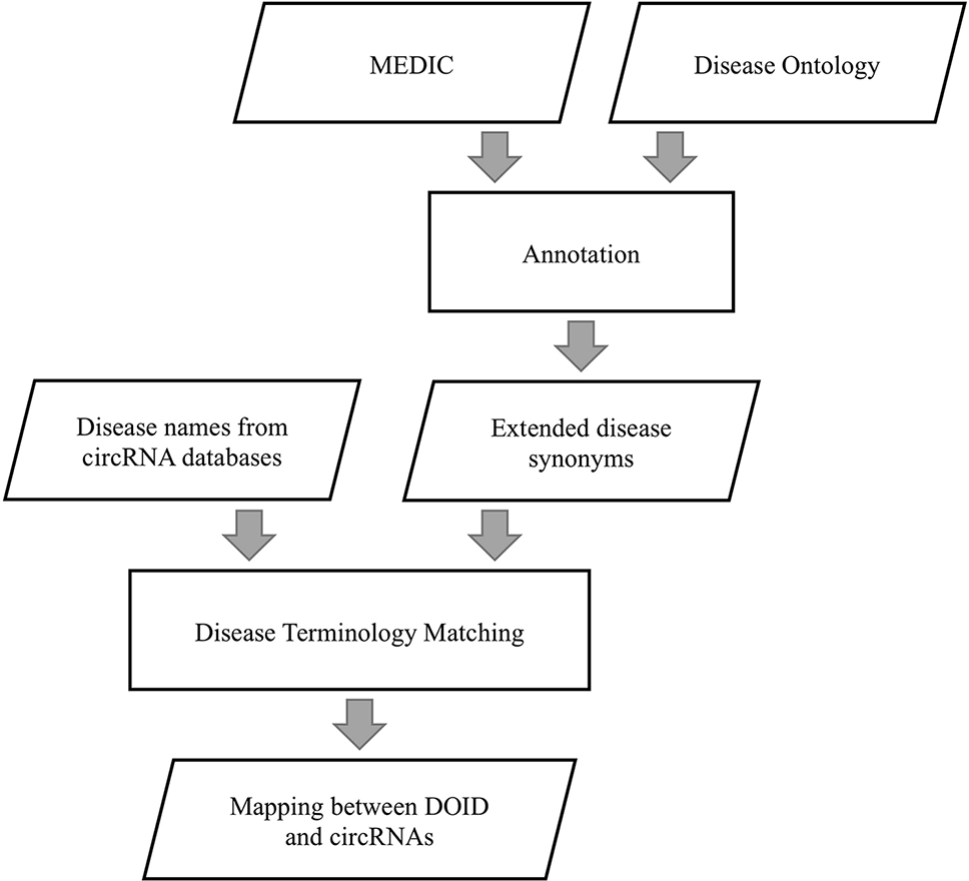
Flowchart of establishing mappings between circRNAs and disease ontology terms.

We extracted disease terms and synonyms from MEDIC to annotate DO by the same external references in DO and MEDIC, as shown in **Figure 2**. If a disease term was recorded in both DO and MEDIC, the term and its synonyms in MEDIC were used to annotate DO. With this approach, a given disease name can be matched to DO to a great extent by string matching, considering that the naming rules for diseases in different disease-related circRNA databases are different. The diseases described by different names are considered to be the same disease that has a unique id in DO if these disease names can match the disease term or its extended synonyms in DO.

### circRNA Expression Similarity

Considering that comprehensive circRNA expression data are still unavailable, we extracted circRNA expression profiles from circBase and CIRCpedia, including the expression profiles of 92488 circRNAs in 78 human cell types or tissues. We used the Spearman correlation coefficient between the expression profiles of each circRNA as the circRNA expression similarity, as shown in Formula 1.

(1) $$\begin{array}{c} \rho =1-\frac{6\sum d_{i}^{2}}{n\left(n^{2}-1\right)} \end{array}$$

where *d_i_* is the difference between the two ranks of the expression scores in the *i*th human cell type or tissue, and *n* is the number of the human cell types or tissues from circBase or CIRCpedia. Matrix *CB* and Matrix *CP* are, respectively, denoted as the circRNA expression similarity matrix of circBase and CIRCpedia, where *CB(i,j)* and *CP(i,j)* are the expression similarities between circRNA *c(i)* and *c(j)*. Then, to obtain reliable performance for circRNA expression data, we defined the expression similarity between circRNA *c(i)* and *c(j)* as shown in Formula 2 if circRNA *c(i)* and *c(j)* are included in both circBase and CIRCpedia.

(2) $$\mathrm{ExSim}\left(i,j\right)=\left\{ \begin{array}{cc} \mathrm{Max}\left(\mathrm{CB}\left(i,j\right),\mathrm{CP}\left(i,j\right)\right) & \mathrm{Max}\left(\mathrm{CB}\left(i,j\right),\mathrm{CP}\left(i,j\right)\right)\geq \tau \\ 0 & \text{otherwise} \end{array}\right.$$

To reduce the impact of data noise, we set a threshold τ to filter out those weak similarities between circRNAs. The threshold τ is set to 0.7 based on our experiments.

### circRNA Functional Similarity

**Figure 3:**
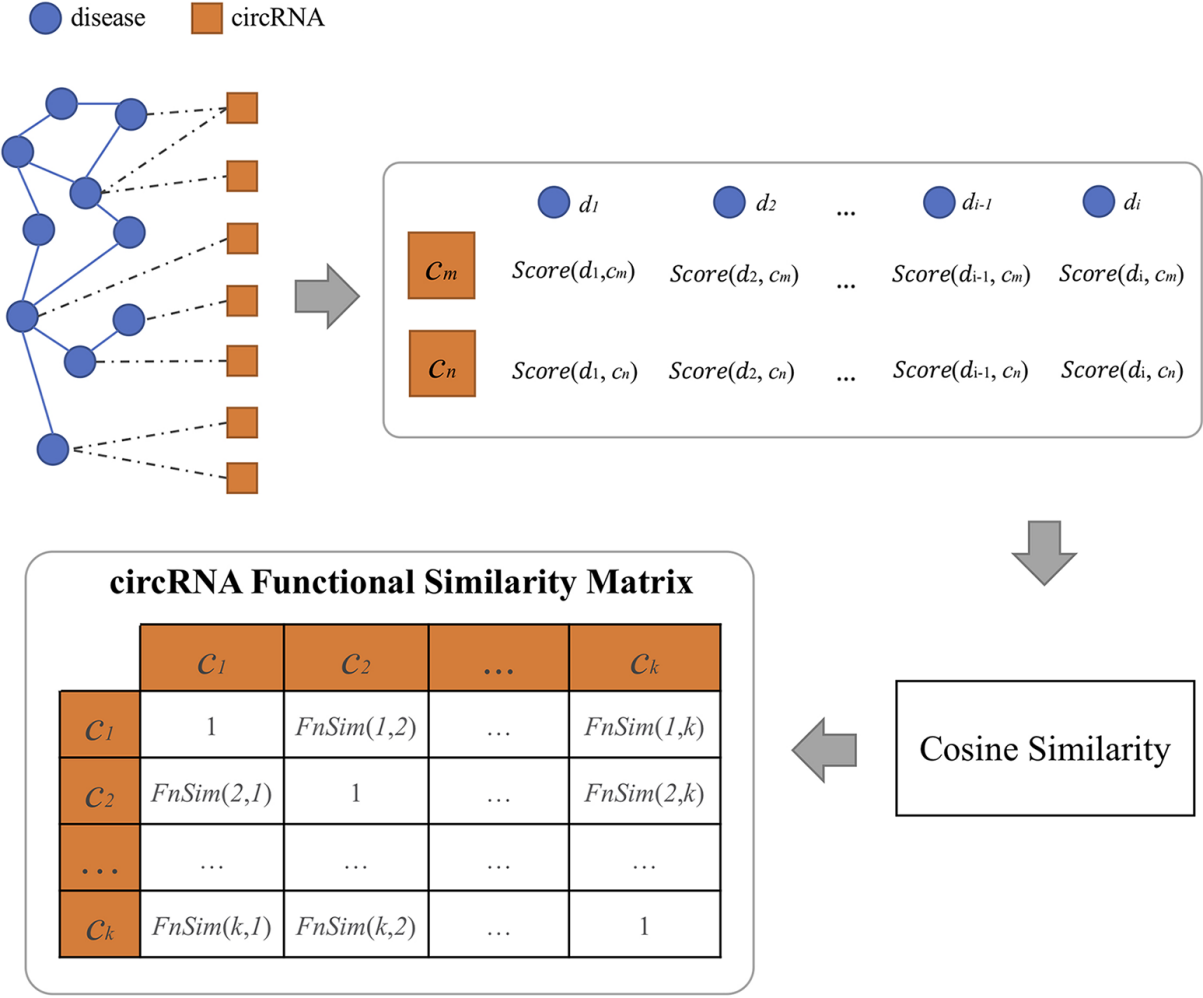
Flowchart of calculating circRNA functional similarity.

We extracted circRNA–disease associations from these above circRNA databases and defined a relational matrix of circRNAs and diseases. For each circRNA, all diseases in the matrix can be used to make a vector in a multidimensional space. Because of the limited number of available disease–circRNA pairs, there is a data sparsity problem in the matrix. Therefore, we calculated the circRNA-related disease similarity and filled this matrix with predicted association scores based on disease–circRNA associations and the disease similarity. Here, we use FNSemSim (Wang et al., 2017) to calculate disease similarity. This method, which combines disease functional similarity and semantic similarity, has good performance for calculating similarities between diseases. The workflow of calculating circRNA functional similarity is shown in **Figure 3**.

To calculate the association between one circRNA and any disease, the similarities between this disease and all diseases that are directly related to this circRNA are calculated by FNSemSim. *C* is defined as the set of disease-related circRNAs, and *D* represents the set of circRNA-related diseases. DisSet(c) is defined as the set of diseases directly related to circRNA *c*. The association score between disease *dis* and circRNA *c* is defined as follows:

(3) $$\begin{array}{c} \mathrm{Score}\left(\text{dis},c\right)=\{\begin{array}{c} \mathrm{Max}\left(\mathrm{FNSemSim}\left(\text{dis},{\text{dis}}_{i}\right)\right)\\ 1 \end{array}\;\;\;\;\begin{array}{c} {\text{dis}}_{i}\in \text{DisSet}\left(c\right),\;\;\;\;\text{dis}\notin \text{DisSet}\left(c\right)\\ \text{dis}\in \text{DisSet}\left(c\right) \end{array} \end{array}$$

where DisSet(c) ⊆D, 1*≤*i *≤* |DisSet(c)|; |DisSet(c)| is denoted as the number of diseases in DisSet(c). If this disease belongs to DisSet(c), the score is 1; otherwise, the score is defined as the maximum of similarities between this disease and all the diseases related to circRNA *c*. Therefore, circRNA *c* can be depicted by a vector that is composed of circRNA-related diseases in a multidimensional space. We can calculate the functional similarity between any two circRNAs based on cosine similarity. The functional similarity between circRNA *c(m)* and *c(n)* is defined as follows:

(4) $$\begin{array}{c} \mathrm{FnSim}\left(m,n\right)=\frac{\sum_{i=1}^{\left|D\right|}\text{Score}\left({\text{dis}}_{i},c\left(m\right)\right)\times \text{Scor}e\left({\text{dis}}_{i},c\left(n\right)\right)}{\sqrt{\sum_{i=1}^{\left|D\right|}\text{Score}{\left({\text{dis}}_{i},c\left(m\right)\right)}^{2}}\sqrt{\sum_{i=1}^{\left|D\right|}\text{Score}{\left({\text{dis}}_{i},c\left(n\right)\right)}^{2}}} \end{array}$$

where |D| represents the size of the circRNA-related disease set *D*, and dis*_i_* is the *i*th disease in the circRNA-related disease set *D*.

### Prediction of Candidate Disease-Related circRNAs

We take circRNA functional similarity and expression similarity as weights to construct a circRNA association network. In this network, the weight between circRNA *c(i)* and *c(j)* is defined as shown in Formula 5. If *ExSim(i,j)* is greater than 0, the weight between circRNA *c(i)* and *c(j)* is the average value of their functional similarity and expression similarity; otherwise, the weight is defined as the functional similarity between them.

(5) $$\begin{array}{c} \mathrm{CircWeight}\left(i,j\right)=\{\begin{array}{c} \left(\text{FnSim}\left(i,j\right)+\text{ExSim}\left(i,j\right)\right)/2\\ \mathrm{FnSim}\left(i,j\right) \end{array}\;\;\;\;\begin{array}{c} \text{if ExSim}\left(i,j\right)>0\\ \text{otherwise} \end{array} \end{array}$$

To predict candidate disease-related circRNAs, the associations between diseases and circRNAs are also considered in this network. The weight between circRNA *c* and disease *dis* is defined as shown in Formula 6. If the disease is directly related to circRNA *c*, the weight between them is 1; otherwise, the weight is 0.

(6) $$\begin{array}{c} \mathrm{CircDisWeight}\left(i,\text{j}\right)=\{\begin{array}{c} 1\\ 0 \end{array}\;\begin{array}{c} \text{if dis}\in \text{DisSet}\left(c\right)\\ \text{otherwise} \end{array} \end{array}$$

In this network composed of circRNAs and diseases, we identify novel candidate disease-related circRNAs based on the PR. PersonalRank algorithm, as a recommendation algorithm based on random walking, can reveal more information between a target node and all the others in a specific network. PersonalRank algorithm is defined as follows:

(7) $$\begin{array}{c} \mathrm{PR}\left(i\right)=\left(1-d\right)r_{i}+d\underset{j\in \text{in}\left(i\right)}{\sum }\frac{\text{PR}\left(j\right)}{\left|\text{out}\left(j\right)\right|} \end{array}$$

where PR(*i*) represents the possibility value that node *i* is accessed; *d* is the transfer probability; out(*j*) represents the out-degree of node *j*; in(*i*) is the in-degree of node *i*; and *r_i_* is defined as follows:

(8) $$\begin{array}{c} r_{i}=\{\begin{array}{c} 1\\ 0 \end{array}\;\;\;\;\begin{array}{c} \text{if}\;i=t\\ \text{if}\;i\neq t \end{array} \end{array}$$

where *t* represents the target node. According to previous studies (Kang et al., 2014; Cheng et al., 2018b), *d* is set to 0.85. The target node *t* in the network randomly moves to adjacent nodes with the probabilities of the edges between these nodes. After enough iterations, the probabilities from the target node to all the other nodes will become stable. Eventually, the algorithm outputs the relevance degrees between all the nodes and this target node.

## Results

### circRNAs and Diseases

**Figure 4:**
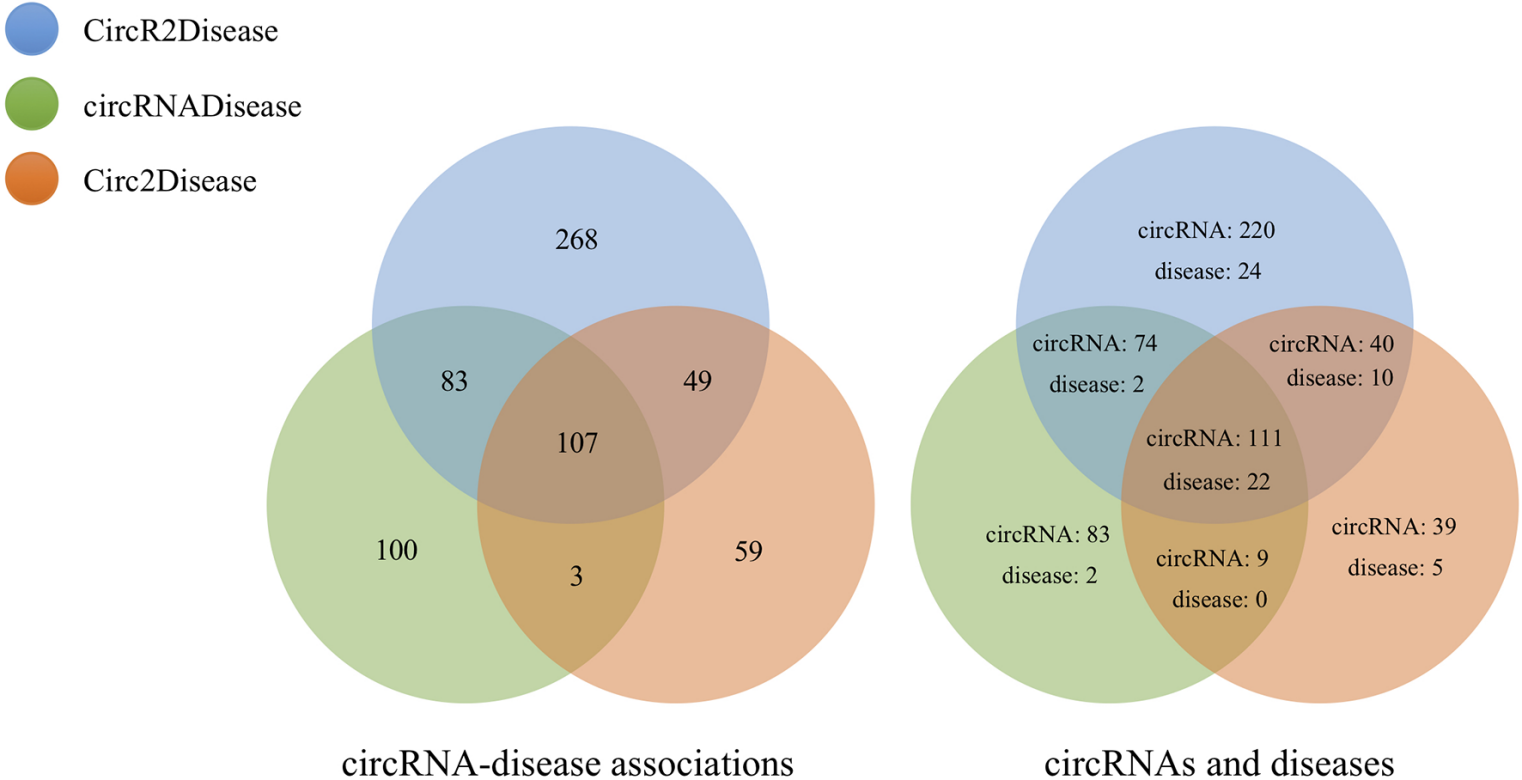
Data distribution in the three databases.

We calculated similarities between 323 circRNAs from circBase and CIRCpedia based on circRNA expression profiles. Then, we obtained 11,281 circRNA pairs based on the preset threshold. Additionally, we found 507 relationships between 58 diseases and 445 circRNAs by mapping DO terms to the diseases in CircR2Disease. We matched 26 diseases based on DO terms and extracted 293 relationships between 277 circRNAs and these diseases from circRNADisease. In Circ2Disease, 218 relationships between 37 diseases and 199 circRNAs were found. Based on DO terms and the unification of circRNA naming, we analyzed the three circRNA databases and found the same circRNAs and diseases among these databases, as shown in **Figure 4**. This provided the test data for the performance evaluation of PreCDA.

We separately calculated the similarities between 445 circRNAs from CircR2Disease, 277 circRNAs from circRNADisease and 199 circRNAs from Circ2Disease. Three circRNA association networks were built that in turn contained 96,580 associations between 440 circRNAs associated with 56 diseases; 38,226 associations between 277 circRNAs associated with 26 diseases; and 18,915 associations between 195 circRNAs associated with 36 diseases. The detailed statistics of the circRNAs and diseases are shown in **Table 1**.

**Table 1:** Information on the three circRNA association networks.

Database	circRNA association network		
	circRNA	Disease	Association
CircR2Disease	440	56	96,580
circRNADisease	277	26	38,226
Circ2Disease	195	36	18,915

### Performance

**Figure 5:**
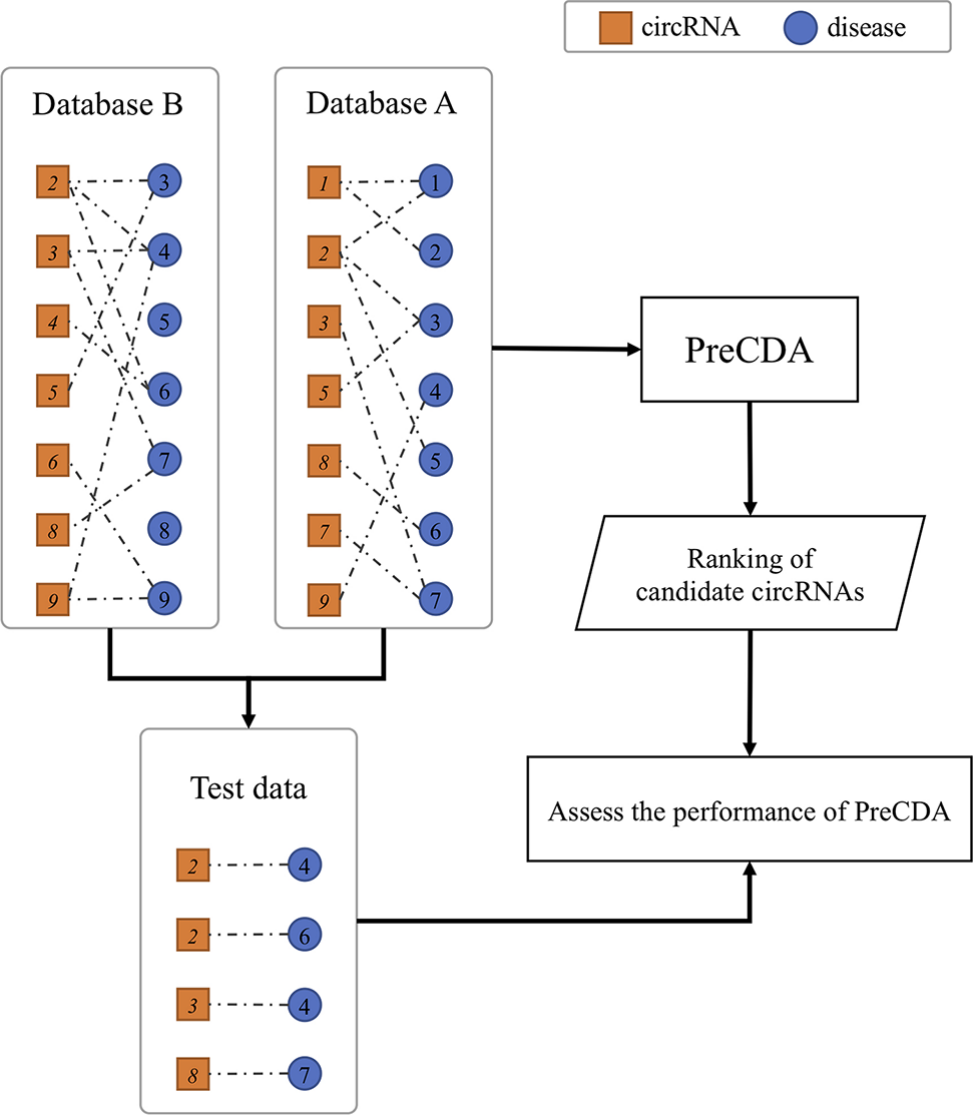
The validation scheme of the computational model. For comparison with database B, test data are extracted from database A according to the test scheme. PreCDA outputs the ranks of candidate circRNAs with the circRNA–disease associations from database A as the input. The performance of PreCDA is assessed based on the test data.

We designed a test scheme to assess the performance of PreCDA. First, we selected two circRNA–disease databases, one to build the circRNA association network and the other to provide test data. Then, we extracted the same diseases from the circRNA association network and the reference database. For a given disease, if any circRNA related to this disease in the reference database exists in the network, but the association between the circRNA and the disease does not, the circRNA can be used as a test case for the disease to assess the performance of this circRNA association network. The test scheme is shown in **Figure 5**.

In this article, we used three circRNA–disease databases, including CircR2Disease, circRNADisease, and Circ2Disease. For example, both circRNA hsa_circ_0000284 and liver cancer (DOID: 3571) were recorded in Circ2Disease and CircR2Disease. The circRNA hsa_circ_0000284 was related to liver cancer (DOID: 3571) in Circ2Disease but not in CircR2Disease. Therefore, we built a circRNA association network based on CircR2Disease and calculated the relevance degrees between liver cancer and all circRNAs unrelated to the disease. We calculated the area under the receiver operating characteristic curve (AUC) according to the ranking of the circRNA hsa_circ_0000284 among these circRNAs to measure the prediction results. To validate the reliability of the computational model, we conducted nine validation experiments based on this scheme involving 18 diseases. We built three circRNA association networks based on the three different circRNA–disease databases. The three data sources were also used as the reference data. Additionally, we merged the known circRNA–disease associations in the three databases as an additional control data source.

**Figure 6:**
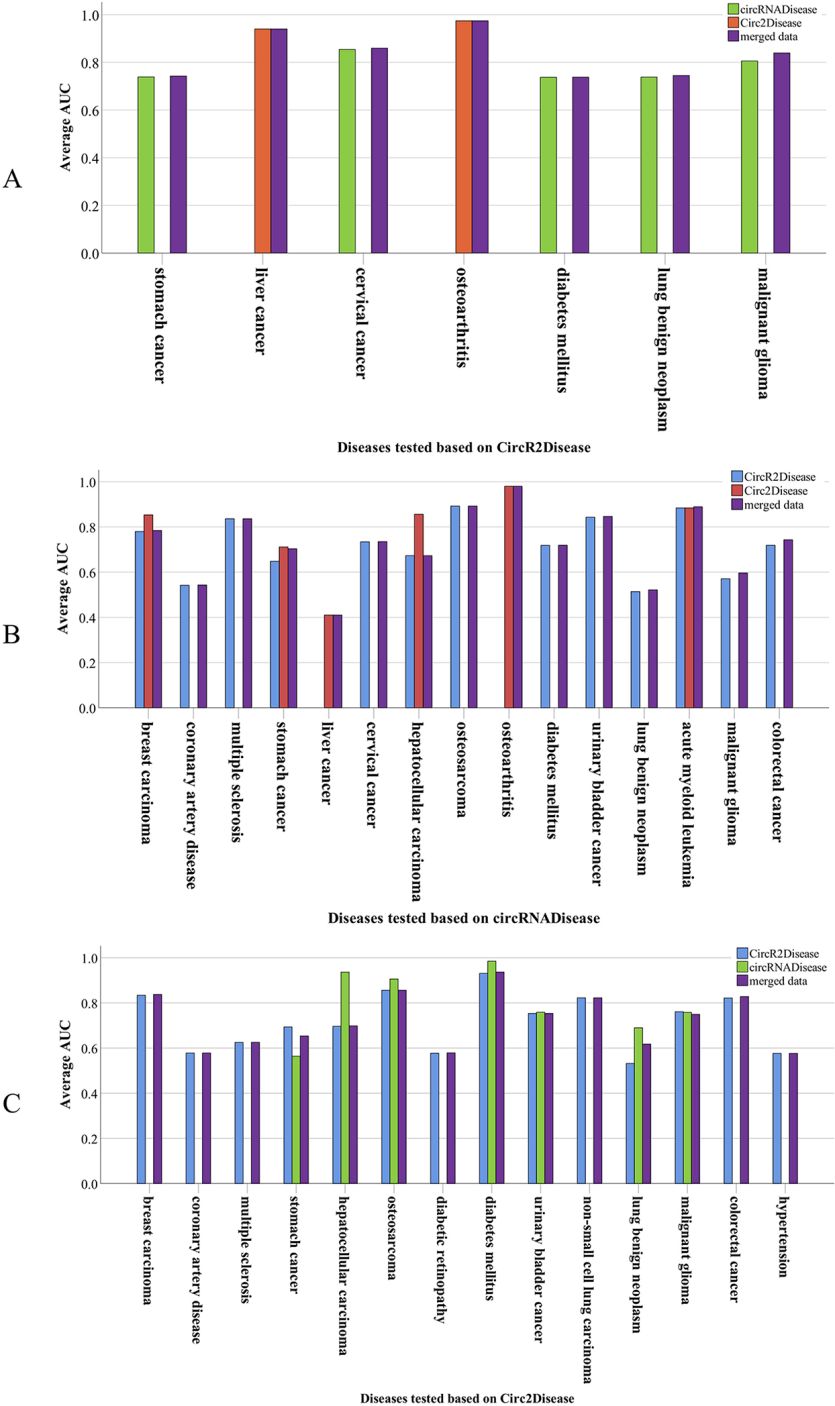
The performance in predicting circRNA-associated diseases. **(A)** Seven diseases were tested based on CircR2Disease with reference to circRNADisease, Circ2Disease, and all circRNA–disease associations from the three data sources. **(B)** Fifteen diseases were tested based on circRNADisease with reference to CircR2Disease, Circ2Disease, and all circRNA–disease associations from the three data sources. **(C)** Fourteen diseases were tested based on Circ2Disease with reference to CircR2Disease, circRNADisease, and all circRNA–disease associations from the three data sources.

PreCDA had an average AUC value of 78.15% in predicting candidate disease-related circRNAs. Furthermore, it had an outstanding performance on some diseases. For example, diabetes mellitus (DOID: 9351) in the network from Circ2Disease had an AUC of 98.48% based on the control data from circRNADisease and an AUC of 93.04% based on the control data from CircR2Disease. Based on the control data from Circ2Disease, the AUC of osteoarthritis (DOID: 8398) was 97.44% in the network from CircR2Disease and 98% in the network from circRNADisease. In the network from Circ2Disease, the AUC of stomach cancer (DOID: 10534) was 56.41% based on the control data from circRNADisease; it had an AUC of 73.88% in CircR2Disease. This shows that the networks from the different data sources have different results for a disease based on the same control database. However, the AUCs in the other validation experiments achieved more than 65%. Even so, the performance of PreCDA is excellent in predicting candidate disease-related circRNAs. The performance of PreCDA based on the different databases and the different control data sources is shown in **Figure 6**.

### Case Study

To further evaluate the performance of PreCDA in predicting potential disease-related circRNAs, we conducted some case studies, including prostate cancer (DOID: 10283), liver cancer (DOID: 3571), breast carcinoma (DOID: 3459), Alzheimer disease, and pancreatic cancer (DOID: 1793). We integrated the known associations between circRNAs and diseases in the three databases and prioritized candidate disease-related circRNAs based on PreCDA.

In the ranking of candidate circRNAs related to liver cancer (DOID: 3571), hsa_circ_0001727 (Qiu et al., 2018) ranked 4th, hsa_circ_0001946 (Yu et al., 2016) ranked 7th, and hsa_circ_0001141 (Guo et al., 2017) ranked 19th. They ranked in the top 3% and were associated with liver cancer. For prostate cancer (DOID: 10283), hsa_circ_0001946 (Zhang et al., 2018) and hsa_circ_0001649 (Yi et al., 2016) ranked 3rd and 5th in the ranking, respectively. They were documented to be related to prostate cancer. For pancreatic cancer (DOID: 1793), CircRNA_100782 (Chen et al., 2017), which ranked 1st in the ranking, was validated to regulate pancreatic carcinoma proliferation through the IL6-STAT3 pathway. We found that some candidate circRNAs related to these diseases were included by Circ2Traits (Ghosal et al., 2013), which is a comprehensive database for circRNAs potentially associated with disease and traits. For example, hsa_circ_0000118, which ranked 1st in the ranking of candidate circRNAs associated with prostate cancer, was documented to be potentially related to this disease in Circ2Traits. The prediction results of the case studies are presented in **Table 2**.

**Table 2:** The prediction results of predicting candidate circRNAs for five diseases.

Disease name	DOID	circRNA	Rank	Evidence
Prostate cancer	10283	hsa_circ_0000118	1	Circ2Traits
		hsa_circ_0001946	3	Zhang et al., 2018
		hsa_circ_0001649	5	Yi et al., 2016
		hsa_circ_0001070	7	Circ2Traits
		hsa_circ_0001512	16	Circ2Traits
		hsa_circ_0000437	18	Circ2Traits
		hsa_circ_0001727	45	Circ2Traits
		hsa_circ_0000130	52	Circ2Traits
Breast carcinoma	3459	hsa_circ_0001070	7	Circ2Traits
		hsa_circ_0001727	19	Circ2Traits
		hsa_circ_0001333	35	Circ2Traits
		hsa_circ_0000190	54	Circ2Traits
Liver cancer	3571	hsa_circ_0001727	4	Qiu et al., 2018
		hsa_circ_0001946	7	Yu et al., 2016
		hsa_circ_0001141	19	Guo et al., 2017
Pancreatic cancer	1793	hsa_circ_0000284	1	Chen et al., 2017
		hsa_circ_0002702	5	Circ2Traits
		hsa_circ_0001667	29	Circ2Traits
Alzheimer disease	10652	hsa_circ_0000284	8	Circ2Traits
		hsa_circ_0001141	28	Circ2Traits
		hsa_circ_0000096	32	Circ2Traits

## Discussion

**Figure 7:**
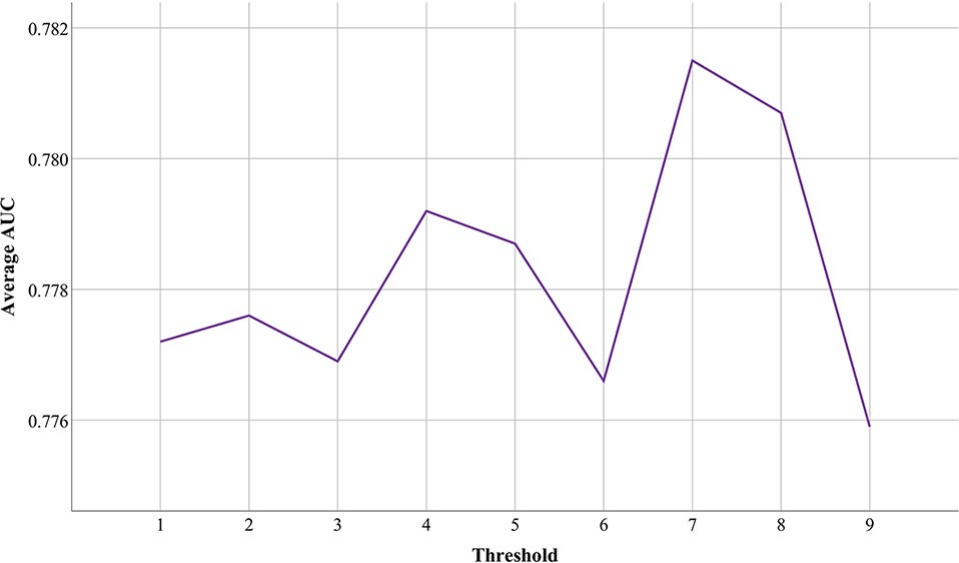
The impact of different thresholds on the performance of PreCDA.

Although functional associations between circRNAs are measured based on circRNA expression profiles, there are many weak connections among them. To reduce the impact of data noise, we set a threshold to filter out those weak connections between circRNAs. Based on the above validation strategy and different thresholds, we conducted nine groups of experiments in which these three databases were used as a reference to each other and to test the performance of PreCDA. As shown in **Figure 7**, the average AUC of PreCDA varied with the change in the threshold, and the computational model worked best when the threshold was set to 0.7.

**Figure 8:**
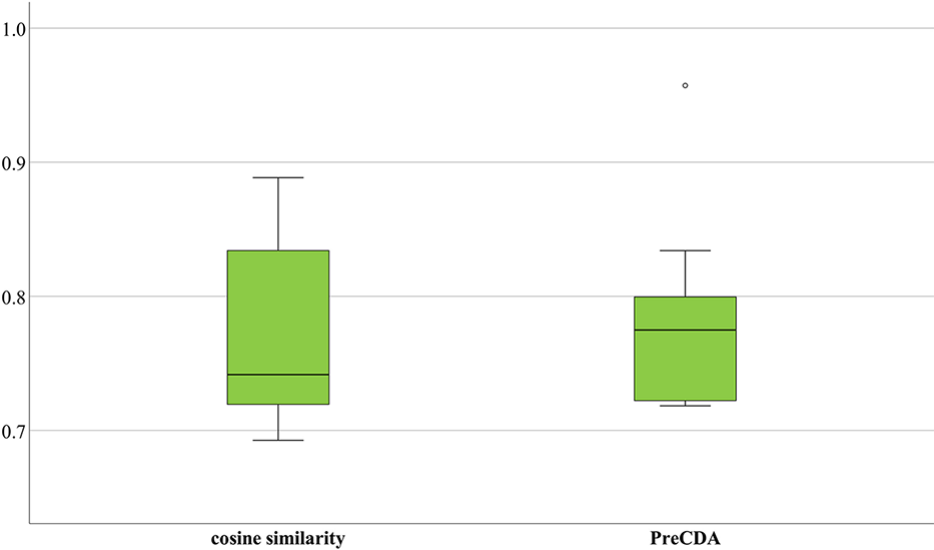
The performance of different computational models.

We calculated circRNA similarities by only cosine similarity and built a circRNA association network. Additionally, we merged the known circRNA–disease associations in these three databases as an additional control data source. Based on the validation strategy mentioned above, we used these three databases to test the performance of the network. As shown in **Figure 8**, the average AUC was 77.22%, the minimum AUC was 69.26%, and the maximum AUC was 88.85%. In comparison, PreCDA has a more stable performance, with an average AUC of 78.15%. Its minimum and maximum AUCs are 71.83% and 95.72%, respectively.

We found that the performance of predicting potential disease–circRNA pairs in the disease-related circRNA association network was impacted by different data sources. The result of predicting the associations between the same diseases and circRNAs was different based on the different data sources that were used to build networks. For example, referring to CircR2Disease, some of the data to be tested in the networks built based on circRNADisease and Circ2Disease were the same. However, the AUC values of predicting the associations between them were different. As shown in **Table 3**, we predicted the associations between colorectal cancer (DOID: 9256) and four circRNAs, including hsa_circ_0001649, hsa_circ_0000284, hsa_circ_0014717, and hsa_circ_0001141. The AUC value for the network of circRNADisease was 71.86%. The performance of identifying the associations between colorectal cancer and these four circRNAs based on Circ2Disease was improved, and its AUC achieved 82.17%.

**Table 3:** Performance differences of predicting circRNA–disease pairs based on different data sources.

References database	Disease	DOID	AUC		circRNA
CircR2Disease			circRNADisease	Circ2Disease	
	Colorectal cancer	9256	71.86%	82.17%	hsa_circ_0001649 hsa_circ_0000284 hsa_circ_0014717 hsa_circ_0001141
	Malignant glioma	3070	57.1%	76.1%	hsa_circ_0000284 hsa_circ_0001649 hsa_circ_0001445
	Lung benign neoplasm	3683	51.4%	53.18%	hsa_circ_0001821 circUBAP2
	Diabetes mellitus	9351	71.85%	93.04%	hsa_circ_0000284
	Coronary artery disease	3393	54.21%	57.78%	hsa_circ_0000615
			CircR2Disease	Circ2Disease	
circRNADisease	Diabetes mellitus	9351	73.73%	98.48%	hsa_circ_0054633
	Malignant glioma	3070	80.6%	75.77%	hsa_circ_0001946 hsa_circ_0004214
			CircR2Disease	circRNADisease	
Circ2Disease	Osteoarthritis	8398	97.44%	98%	hsa_circ_0000026

## Conclusions

Circular RNA plays an important role in the development of various pathological conditions. Research on circRNA is invaluable in explaining the underlying pathogenesis. Therefore, we proposed a computational model to identify candidate disease-related circRNAs. First, we calculated the circRNA expression similarity with the circRNA expression profiles. Then, the disease similarity was used as dimensions of circRNA vectors, and the circRNA functional similarity was calculated based on cosine similarity. We defined the associations between circRNAs and diseases based on the circRNA expression similarity and functional similarity. A disease-related circRNA association network was built, and potential candidate disease-related circRNAs were ranked by the PR.

We evaluated the performance of PreCDA with the help of data differences among these three databases, including CircR2 Disease, circRNADisease, and Circ2Disease. The results showed that the average AUC of PreCDA was 78.15%, and it had good performance in predicting potential disease-related circRNA signatures. We discussed the selection of the threshold and the impact of different data sources on the performance of PreCDA. Then, we used several common diseases as case studies and found some unknown circRNAs that could be related to these diseases based on PreCDA. The findings of this study could be further applied in analyzing diseases in a system biology perspective (Cheng and Hu, 2018) and helpful for researchers to improve disease diagnostics and treatments.

## Data Availability

PreCDA is implemented using a combination of Java and scala, and it is freely available from the website at https://github.com/wythit/PreCDA.

## Author Contributions

YoW and CN did data collection and preprocessing. And with the guidance of TZ and YaW, YoW finished the algorithm design and validation. YoW was the major contributor in writing the manuscript. All authors have read and approved the final version of the manuscript.

## Funding

Publication costs were funded by the National Key Research and Development Program of China (grant no. 2016YFC0901605, 2016YFC1201702-01) and the National High-tech R&D Program of China (grant no. 2012AA02A604, 2015AA020108).

## Conflict of Interest Statement

The authors declare that the research was conducted in the absence of any commercial or financial relationships that could be construed as a potential conflict of interest.
